# Assessment of Patient Experiences in an Academic Hospital in Saudi Arabia

**DOI:** 10.7759/cureus.24203

**Published:** 2022-04-17

**Authors:** Diyaa H Bokhary, Omar M Saggaf, Ayman M Baabdullah, Yousof O Kabli, Kamal W Ghalayieni

**Affiliations:** 1 Emergency Medicine, King Abdulaziz University Hospital, Jeddah, SAU; 2 Internal Medicine, King Abdulaziz University Hospital, Jeddah, SAU; 3 Orthopedic Surgery, International Medical Centre Hospital, Jeddah, SAU; 4 Pediatrics, King Abdulaziz University Hospital, Jeddah, SAU; 5 Cardiology, King Abdulaziz University Hospital, Jeddah, SAU

**Keywords:** medical services, patient needs, patient satisfaction, health care, department of medicine

## Abstract

Introduction

Patient continuous assessment is an important component of patient-centered healthcare systems and requires the identification of the services and resources of these systems to ensure patient satisfaction. This study aimed to determine the factors that affect patient satisfaction, identify patients' unmet health care and informational needs, and suggest measures to fill these gaps in healthcare systems.

Methods

A cross-sectional study included 235 patients who were admitted to the medical ward of an educational tertiary healthcare center in Jeddah, Saudi Arabia, between June-September 2016. A self-administered questionnaire based on the Arabic version of the "échelle de qualité des soins en hospitalisation" or the quality of care in hospitalization scale (ESQ-H) consisting of two subscales that measured their satisfaction with the services received was offered to the study participants. We analyzed the data to identify factors associated with patient dissatisfaction using IBM SPSS® Statistics Version 21.0.

Results

The patients included 145 males (61.7%) and 90 females (38.3%). The Cronbach's alpha coefficient was 0.933 for the questionnaire. In the subscale associated with the quality of the medical information patients received, three independent variables were associated with higher patient satisfaction: planned patients' hospital stay (p<0.001), patients' health improvement (p<0.001), and patients' overall life satisfaction (p<0.001). In the subscale associated with patients' relationship with medical staff and the daily routine of the medical ward, four independent variables were significant: male gender (p=0.007), patients, if the hospital stay was planned (p=0.009), improvement of patients' health (p<0.001), and patients' overall life satisfaction (p=0.006).

Conclusion

Patients' satisfaction level was "very good" with the medical information subscale and "excellent" with the relationship subscale. We found that although patients were satisfied with some aspects of their health care, other aspects required more attention; hence, the resolution of patients' unmet health care and informational needs should be prioritized by stakeholders to improve patient satisfaction. Furthermore, the patients should be informed about what they can expect during their upcoming hospital stay, their overall life satisfaction should be considered, and patients with issues related to their life satisfaction should be appointed a social worker.

## Introduction

The measurement of health care quality has become increasingly more prevalent among both healthcare providers and purchasers as patients have become more knowledgeable consumers of health care [1]. Identification and fulfillment of patients' needs is an important component of patient-centered healthcare systems [2], as performance and quality of health care are commonly measured by patient satisfaction. In a competitive healthcare system, long-term organizational survival depends on high customer retention and low attrition - satisfied patients are likely to be loyal customers of the hospital and recommend it to others, whereas unsatisfied customers may instead seek healthcare organizations that outperform their current service providers [3]. Hence, meeting patients' expectations requires the identification of the services and resources of these healthcare systems to determine the factors that affect patient satisfaction, identify patients' unmet health care and informational needs, and ensure patient satisfaction [4].

A survey conducted by Mahatma Gandhi Medical College and Research Institute in Puducherry, India, observed that patients' satisfaction was influenced by the efficiency of the hospital admissions process, the performance of the medical records department, pharmacy services, pantry services, and patient's perceived level of care provided by nurses and physicians. The study findings revealed that patients' satisfaction was not influenced by the age of the patients, hospital location, qualification, duration of hospital stay and cleanliness [5]. A similar study done in a Moroccan hospital had positive feedback about the quality of their services through measuring patient satisfaction, but, importantly, also identified specific areas of improvement to increase patient satisfaction [6].

Continuous monitoring and evaluation of the quality of health care and patients' satisfaction are crucial to the identification of gaps in the delivery of healthcare services. In our literature review, we found scant research about health care quality and patients' satisfaction in the Kingdom of Saudi Arabia (Saudi Arabia) or in the Middle East in general. In this study, we focused on patients who were discharged from the medical ward of King Abdulaziz University Hospital (KAUH), a teaching hospital in Jeddah, Saudi Arabia, and aimed to determine the factors that affect patients' satisfaction, identify patients' unmet health care and informational needs, and suggest measures to fill these gaps in health care to improve patients' satisfaction.

## Materials and methods

Our cross-sectional study observed a sample of 235 patients who were discharged from the medical ward of KAUH between June 1­ and September 1, 2016. After being discharged, patients were contacted by phone. Patients who died and patients who spent less than 24 hours in the medical ward were excluded. After collecting the patients' personal contact information from hospital records, data collectors contacted the patients by phone. After obtaining informed verbal consent, the patients were asked to complete a questionnaire about their hospital stay. All communications and personal data were kept anonymous and confidential. No personal data was required for data analysis and results.

The data was collected utilizing a self-administered questionnaire based on the Arabic version of a widely used scale called the EQS-H (échelle de qualité des soins en hospitalisation or the quality of care in hospitalization scale) to assess patients' satisfaction with medical and nursing care from intake through discharge. The construct of patient satisfaction included two dimensions measured by subscales: the quality of the medical information provided to the patients (MI) and the quality of the patients' relationship with physician and nursing staff and the daily routine of the medical ward (RS). The questionnaire consisted of 16 items, with two subscales (i.e., MI and RS) with eight items in each. The patients indicated the extent of their agreement or disagreement with each item, and each of the 16 items was rated on a five-point scale of satisfaction ranging from one (poor) to five (excellent) [6]. The degree of satisfaction was calculated as the sum of scores of the eight items on each subscale, and the global score was the sum of both subscales. Thus, the score on each subscale can range from 8 to 40, and the total score can range from 16 to 80 (i.e., 16 represents the lowest possible rating of satisfaction and 80 represents the highest possible rating of satisfaction) [7]. In addition, information regarding demographic, socioeconomic, and health characteristics was obtained from the patients.

IBM SPSS Statistics Version 21.0 (IMB Inc., Armonk, USA) was used for data entry and analysis. Data was expressed as mean and standard deviation for continuous data and frequency and percentage for categorical data. Cronbach's alpha reliability coefficient was used to assess the internal consistency of the EQS-H questionnaire items. An acceptable alpha coefficient was considered to be >0.70 [8,9]. The Student t-test was used to evaluate the statistical difference between two groups and the analysis of variance (ANOVA) test for more than two groups. The relationship between the two dimensions of patients' satisfaction (i.e., MI and RS) and patients' overall life satisfaction was described by the Pearson correlation coefficient (r). Variables with a p-value of 0.25 or less were included in the multivariate analysis. Multivariate analysis was conducted using multiple linear regression. A two-tailed p-value of <0.05 was considered statistically significant.

## Results

Characteristics of the sample 

The sample consisted of 235 patients discharged from KAUH that completed a questionnaire based on an Arabic version of the EQS-H, among which 145 (61.7%) were male and 90 (38.3%) were female. The patients' demographic, socioeconomic, and health characteristics are outlined in Table 1.

**Table 1:** Univariate analysis of predictors of satisfaction related to demographic and socioeconomic characteristics MI - quality of medical information; RS - relationship with staff and daily routine; KAUH - King Abdulaziz University Hospital

Characteristics	N (%)	MI		RS	
Gender		Mean satisfaction score	Standard deviation	Mean satisfaction score	Standard deviation
Male	145 (61.7%)	27.4	1.4	32.8	1.3
Female	90 (38.3%)	27.0	1.6	29.8	1.5
p-value		0.735		0.009	
Age					
<25	47 (20.0%)	28.0	1.8	31.3	1.7
25-39	27 (11.5%)	27.1	1.9	31.6	1.8
40-59	70 (29.8%)	27.8	1.6	31.7	1.6
≥60	91 (38.7%)	25.8	1.6	30.6	1.6
p-value		0.318		0.824	
Occupation/income					
Unemployed or retired	121 (51.5%)	26.6	1.4	31.1	1.3
Less than 10,000 SAR per month	106 (45.1%)	27.5	1.5	29.9	1.4
More than 10,000 SAR per month	8 (3.4%)	27.4	2.7	32.9	2.6
p-value		0.769		0.413	
Residence					
Near KAUH (less than 20 min from hospital)	82 (34.9%)	27.4	1.4	31.4	1.3
In Jeddah (20-40 min from the hospital)	137 (58.3%)	27.7	1.3	32.6	1.3
Outside of Jeddah (more than 40 min from the hospital)	16 (6.8%)	26.5	2.3	29.9	2.2
p-value		0.777		0.228	
Educational level					
Less than high school	129 (54.9%)	28.0	1.5	31.6	1.5
High school	48 (20.4%)	25.8	1.7	31.0	1.6
Tertiary	58 (24.7%)	27.8	1.6	31.3	1.5
p-value		0.210		0.889	
Health insurance					
Yes	45 (19.1%)	27.8	1.6	31.8	1.6
No	190 (80.9%)	26.6	1.4	30.8	1.3
p-value		0.356		0.384	
Nationality					
Saudi	138 (58.7%)	25.7	1.4	29.8	1.3
Non-Saudi	97 (41.3%)	28.7	1.6	32.8	1.5
p-value		0.002		0.002	
Marital status					
Married	167 (71.1%)	27.5	1.5	31.4	1.4
Single	68 (28.9%)	26.9	1.6	31.2	1.6
p-value		0.698		0.913	

Reliability of the EQS-H questionnaire

Cronbach's alpha coefficients were calculated to check the internal consistency of the questionnaire. The Cronbach's alpha for MI was 0.892; for RS, it was 0.902, and for both subscales combined, it was 0.933.

Descriptive statistics of the subscales

In general, the mean value of the total score of the EQS-H was 65.4±15.2 (range: 19-79). The highest mean value (4.6) was observed for one of the items of the RS subscale - efforts to ensure the patient's privacy. Specifically, the mean score of the MI subscale was 31.11±8.8 (range: 8-40), and the mean value for the RS subscale was 34.3±7.6 (range: 8-40).

Overall satisfaction

Patients' satisfaction rate was "very good" on the MI subscale and "excellent" on the RS subscale of the questionnaire, which reflects the high overall satisfaction of the patients with the hospital's health care and informational provisions.

Univariate Analysis

Concerning univariate analysis, statistical differences between groups were evaluated by the Student t-test for comparison between two groups and by one-way analysis of variance (ANOVA) for three groups and above. The Pearson correlation coefficient (r) was calculated to describe the relationships between overall life satisfaction and MI and RS (i.e., the two subscales of patient satisfaction). Variables with p≤0.25 in univariate analysis were selected for inclusion in a multivariate analysis. A two-tailed p<0.05 was considered statistically significant. The results of the univariate analysis are shown in Table 2.

**Table 2:** Univariate analysis of some general predictors of patient satisfaction MI - quality of medical information; RS - relationship with staff and daily routine

Characteristics	N (%)	MI		RS	
Was your hospital stay planned?		Mean satisfaction score	Standard deviation	Mean satisfaction score	Standard deviation
Yes	172 (73.2%)	30.2	1.4	32.2	1.3
No	63 (26.8%)	24.2	1.6	30.4	1.5
p-value		<0.001		0.111	
Did your health get better?					
Yes, it got better	136 (57.9%)	31.1	1.5	34.5	1.4
Some improvement	67 (28.5%)	28.1	1.6	32.5	1.5
No, it got worse	32 (13.6%)	22.4	1.7	26.8	1.7
p-value		<0.001		<0.001	
The quality of physician care or other services provided					
Quality of physician	152 (64.7%)	27.0	1.3	31.6	1.3
Quality of other services provided	31 (13.2%)	27.6	1.8	31.6	1.7
Both	52 (22.1%)	26.9	1.7	30.7	1.6
p-value		0.895		0.702	
Overall life satisfaction					
r		0.225		0.181	
p-value		0.001		0.006	

Determinants of satisfaction

Univariate analysis of the selected variables showed their relationship with the scores related to patient satisfaction. Table 2 presents the summary results of the univariate analysis.

Quality of Medical Information (MI)

Univariate analysis shows that higher satisfaction on the MI subscale was associated with participants who indicated that their hospital stay was planned (p<0.001), their health improved (p<0.001), non-Saudi participants (p=0.002), and overall life satisfaction (p=0.001).

Relationship with Staff and Daily Routine (RS)

Univariate analysis shows that higher satisfaction on the RS subscale was associated with male gender (p=0.009), participants who indicated that their health improved (p<0.001), non-Saudi participants (p=0.002), and overall life satisfaction (p=0.006).

Factor analysis

**Figure 1:**
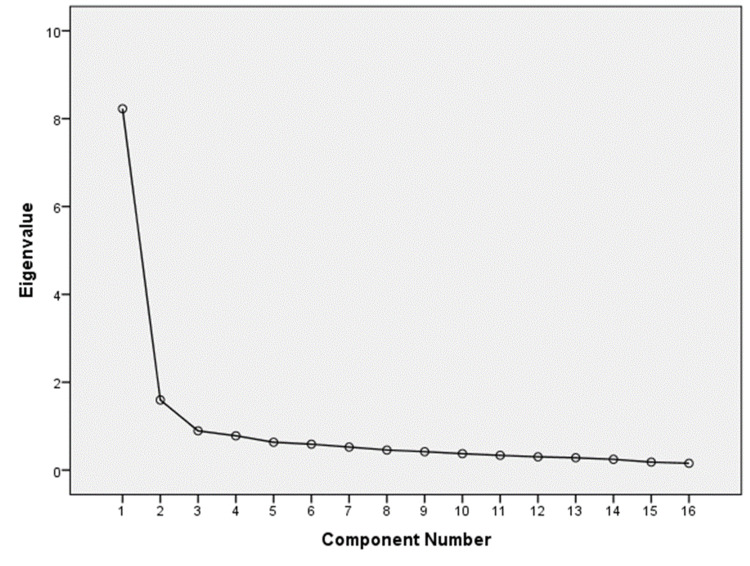
Screen plot of the factor analysis results

Factor analysis is a statistical method used to study the dimensionality of a set of variables. In factor analysis, latent variables represent unobserved constructs and are referred to as factors or dimensions. Factor analysis is an effective method of decreasing a large number of variables, and of limiting the number of factors that reflect the common variance in the variables. The results of factor analysis show the following results. The Kaiser-Meyer-Olkin measure of sampling adequacy equals 0.929, which is greater than 0.5. This reflects the high reliability of factors extracted from factor analysis and means that the sample size is sufficient for conducting factor analysis. Extraction sums of squared loadings before rotating the two factors is 61.39, i.e., the two factors explain 61.39% of the total variance. After rotation, the first factor explains 31.11% of the total variance, and the second factor explains 30.28% of the total variance. The screen plot shows the two extracted factors and the eigenvalues (Figure 1). The first factor reflects the MI dimension, and the second factor reflects the RS dimension (Table 3).

**Table 3:** Results of factor analysis of EQS-H questionnaire MI - quality of medical information; RS - relationship with staff and daily routine; EQS-H - "échelle de qualité des soins en hospitalisation" or the quality of care in hospitalization Extraction method: principal component analysis; rotation method: varimax with Kaiser normalization

Dimension/item	Factor 1	Factor 2
MI		
Clarity of diagnosis explanation	0.746	
Required examinations	0.709	
Examination results	0.662	
Aims of treatment	0.734	
Treatment side effects	0.702	
Future symptoms	0.739	
Future activities	0.649	
Medical care after discharge	0.586	
RS		
Doctor in charge		0.63
Efforts to ensure my privacy		0.691
Assistance was given for activities		0.752
Assistance for pain relief		0.7
Promptness of nurses		0.845
Organization of the ward		0.74
Atmosphere of the ward		0.758
Readiness of nurses to spend time with me		0.821
Percentage of explained variance	31.11	30.28
Mean ± standard deviation	31.12±8.8	34.25±7.6
Cronbach's alpha	0.892	0.902

Table 4 represents the distribution of patients' replies for each item in the EQS-H questionnaire. The results appear in frequencies (n) and percentages (%) for each item over the five levels of response ranging from one (poor) to five (excellent).

**Table 4:** Distribution of the replies on EQS-H questionnaire MI - quality of medical information; RS - relationship with staff and daily routine; EQS-H - "échelle de qualité des soins en hospitalisation" or the quality of care in hospitalization

		Poor	Average	Good	Very good	Excellent
MI						
Clarity of diagnosis explanation	N	22	13	31	33	136
	%	9.4	5.5	13.2	14.0	57.9
Required examinations	N	17	17	20	33	148
	%	7.2	7.2	8.5	14.0	63.0
Examination results explanation	N	27	17	23	26	142
	%	11.5	7.2	9.8	11.1	60.4
Aims of treatment	N	20	12	26	32	145
	%	8.5	5.1	11.1	13.6	61.7
Treatment side effects	N	48	18	25	22	122
	%	20.4	7.7	10.6	9.4	51.9
Future prognosis explanation	N	39	27	27	31	111
	%	16.6	11.5	11.5	13.2	47.2
Expected level of activity participation ability	N	70	17	22	29	97
	%	29.8	7.2	9.4	12.3	41.3
Medical care after discharge	N	22	12	19	36	146
	%	9.4	5.1	8.1	15.3	62.1
RS						
Doctor in charge	N	21	7	20	28	159
	%	8.9	3.0	8.5	11.9	67.7
Efforts to ensure my privacy	N	9	5	14	25	182
	%	3.8	2.1	6.0	10.6	77.4
Assistance given for activities	N	20	9	26	27	153
	%	8.5	3.8	11.1	11.5	65.1
Assistance for pain relief	N	21	10	22	26	156
	%	8.9	4.3	9.4	11.1	66.4
Promptness of nurses	N	19	12	26	32	146
	%	8.1	5.1	11.1	13.6	62.1
Organization of the ward	N	16	5	19	37	158
	%	6.8	2.1	8.1	15.7	67.2
Atmosphere of the ward	N	16	5	28	33	153
	%	6.8	2.1	11.9	14.0	65.1
Readiness of nurses to spend time with me	N	23	8	18	34	152
	%	9.8	3.4	7.7	14.5	64.7

Multivariate analysis

In our study, we elaborated on the effects of various demographic, socioeconomic, and health characteristics (i.e., those with p<0.25) on the satisfaction scores after adjustment for all variables in a multivariate model. Table 5 shows the results of the multivariate analysis.

**Table 5:** Multivariate analysis of relevant variables MI - quality of medical information; RS - relationship with staff and daily routine; KAUH - King Abdulaziz University Hospital

Variables	MI		RS	
Gender	Β Coef.	p-value	Β Coef.	p-value
Male	1.393	0.144	2.403	0.007
Female	0	-	0	-
Was your hospital stay planned?				
Yes	7.035	<0.001	2.758	0.009
No	0	-	0	-
Has your health improved?				
Yes	9.376	<0.001	8.389	<0.001
A little	5.931	<0.001	5.940	<0.001
No	0	-	0	-
Residence				
Near KAUH (less than 20 min from the hospital)	1.137	0.549	1.007	0.570
In Jeddah (20-40 min from the hospital)	0.922	0.617	2.061	0.233
Outside of Jeddah (more than 40 min from the hospital)	0	-	0	-
Educational level				
Less than high school	-1.112	0.317	-0.448	0.666
High school	-2.190	0.108	-0.628	0.621
Tertiary	0	-	0	-
Nationality				
Saudi	-2.796	0.003	-2.864	0.001
Non-Saudi	0	-	0	-
Overall life satisfaction	0.852	<0.001	0.589	0.006

Quality of Medical Information (MI)

Table 5 shows that three independent variables were associated with higher satisfaction on the MI subscale: planned hospital stay (p<0.001); improvement of health (p<0.001); and overall life satisfaction (p<0.001).

Relationship with Staff and Daily Routine (RS)

Table 5 shows that four independent variables were associated with higher satisfaction on the RS subscale: male gender (p=0.007), planned hospital stay (p=0.009), improvement of health (p<0.001), and overall life satisfaction (p=0.006).

## Discussion

Our study utilized an Arabic version of the EQS-H questionnaire to measure patients' satisfaction in the medical ward at King Abdulaziz University in Jeddah. The widely used EQS-H questionnaire was one of several surveys developed and validated in France to measure patients' satisfaction [9]. The EQS-H questionnaire was first translated into Arabic for a study conducted in Morocco in which the psychometric properties of the scale were tested, and the scale showed excellent reliability [6]. In our study, the EQS-H questionnaire showed good reliability. Factor analysis confirmed the bidimensionality of the variables.

Scholars have studied patients' satisfaction with health care in various institutions throughout Saudi Arabia. However, compared to the results of these studies, the overall satisfaction rate was higher in our population [10-19]. Patients' satisfaction rate was "very good" on the MI dimension and "excellent" on the RS dimension of the EQS-H questionnaire. Regarding the overall care, most of the participants were satisfied. A previous similar study showed that patients' satisfaction rate was high regarding their overall care [19].

One of the demographic variables in which a positive relationship was found with patients' satisfaction on both dimensions (i.e., MI and RS) was health improvement. This was in accordance with the findings of other studies. Researchers in Korea found the positive effect of physician empathy on patients' satisfaction and compliance and the positive effect of increased compliance on patient health [20]. A study in the United States also related patients' satisfaction and positive health outcomes to patient-doctor communication [21], and researchers in Italy considered the relationship between the disease complications of diabetic patients and physician empathy and concluded that physician empathy is significantly associated with clinical outcomes [22].

Another variable that affected patients' satisfaction on both the MI and the RS subscales was "hospital stay was planned". This could be because patients whose hospital stay was planned were psychologically and socially better prepared for their stay compared to those whose hospital stay was unplanned. Furthermore, patients' overall life satisfaction was significantly associated with the items on both the MI and the RS subscales. According to the findings of previous studies, informing patients about their care management plans, considering their preferences and needs, and demonstrating an understanding of their problems may improve patients' overall satisfaction [17].

Conversely, our multivariate analysis demonstrated that men are more satisfied than women on the items related to the RS dimension, and this is in accordance with the findings of studies conducted in Vietnam and the United Kingdom [2,23]. However, the findings of other studies disagree with our findings [24]. We posit that this may be due to the gendered Saudi healthcare system and the differential treatment of women as both practitioners and consumers of healthcare: in Saudi Arabia, gender-same nurses work in the segregated women's and men's wards. Moreover, KAUH rotates three shifts with different nurse teams in each ward, and each shift increases the chance of bias.

In this study, we focused on the medical ward of KAUH to determine patients' satisfaction levels for this essential department only. A similar study conducted in Riyadh, Saudi Arabia, concluded that the assessment of the services of hospital departments should be conducted individually since the rate of patients' satisfaction tends to skew more positive if the hospital departments are evaluated as a whole [13].

Limitations

As for any other research, this study had some limitations. First of all, this was a single-center retrospective study conducted among patients admitted to the internal medicine ward, which limits results generalization. Second, although we tried our best to make sure that all patients had a good understanding of the questions, and we double-checked what patients meant by any single answer, patient's opinions and experiences were obtained through regular verbal phone calls after they were discharged home, which may affect their understanding of the questions and the way of expression of their feelings.

Implications for future research

Based on our findings, we encourage future researchers to conduct studies of different hospital departments to identify patients' unmet health care and informational needs, to improve healthcare services and patients satisfaction. We recommend continuous assessment and data collection to identify and improve areas of weakness and evaluate progress.

The highest mean value in our study was the item of "ensuring patient privacy", which received positive feedback from the patients in our study. This high mean was in accordance with another local study conducted at King Khalid University Hospital in Riyadh, Saudi Arabia. This may indicate that physicians in Saudi Arabia are becoming more sensitive to Saudi cultural issues [12], which may result in improved health care and patients satisfaction.

Finally, our research findings support the Saudi Arabia government's transformative Vision 2030 plan that seeks to increase customer-centric behavior and improve client satisfaction in every organization in the country, including healthcare organizations.

## Conclusions

The patient needs assessment is an important component of patient-centered healthcare systems and requires the identification and improvement of the services and resources of these systems to ensure patients satisfaction. Hence, the resolution of patients' unmet health care needs should be prioritized by stakeholders to improve patient satisfaction. Furthermore, patients should be informed about what they can expect during their upcoming hospital stays and their ongoing care management. Their overall life satisfaction should also be considered, and patients with issues related to their life satisfaction should be appointed a social worker.
